# Impact of Physical Activity on Health Behavior Change and Mental Health During the COVID-19 Epidemic Among Chinese Adults: China Health and Retirement Longitudinal Study (CHARLS)

**DOI:** 10.3390/ijerph22020201

**Published:** 2025-01-30

**Authors:** Wupeng Yin, Niliarys Sifre-Acosta, Daisy Chamorro, Susmita Chowdhury, Nan Hu

**Affiliations:** 1 Department of Biostatistics, Robert Stempel College of Public Health and Social Work, Florida International University, Miami, FL 33199, USA; wyin@fiu.edu (W.Y.); dcham034@fiu.edu (D.C.); 2 Department of Dietetics and Nutrition, Robert Stempel College of Public Health and Social Work, Florida International University, Miami, FL 33199, USAschow046@fiu.edu (S.C.); 3 Department of Family and Preventive Medicine, University of Utah School of Medicine, Salt Lake City, UT 84132, USA

**Keywords:** physical activity, health behavior, mental health, older adults, COVID-19

## Abstract

Background: The COVID-19 pandemic caused significant disruptions to daily life, affecting regular physical activity (PA) and health behaviors worldwide. This study investigates the associations between PA domains and changes in health behaviors and mental health outcomes among middle-aged and old Chinese adults. Methods: Using wave 5 cross-sectional data from the 2020 China Health and Retirement Longitudinal Study, we analyzed 17,180 adults aged 45 and above, focusing on health behavior changes such as smoking, alcohol consumption, dietary adjustments, and panic purchasing, as well as mental health outcomes like anxiety and fear. PA was classified by intensity levels—light, moderate, and vigorous—and by activity purposes—total, leisure, and occupational. Results: The findings indicate that leisure PA is associated with healthier behaviors, including lower odds of increased smoking (OR = 0.71, 95% CI: 0.57–0.90) and alcohol consumption (OR = 0.70, 95% CI: 0.54–0.90), whereas occupational PA is linked to adverse behavioral outcomes, such as higher odds of smoking (OR = 1.45, 95% CI: 1.15–1.83) and alcohol use (OR = 1.43, 95% CI: 1.10–1.86). Additionally, participants engaged in all domains of PA were more likely to experience anxiety and fear compared to those who were physically inactive. Conclusions: Our limited understanding of the role PA has on behavioral and mental health during public health crises highlights the importance of having tailored strategies to enhance resilience in similar future scenarios.

## 1. Introduction

The COVID-19 pandemic profoundly impacted global public health [1], disrupting daily routines and posing challenges to maintaining physical activity (PA) and mental well-being [2,3,4,5]. PA is well-documented to improve physical health and reduce the risk of chronic diseases while also mitigating mental health issues, such as depression and anxiety [6,7,8]. These benefits underscore PA’s critical role in protecting health during public health crises like COVID-19.

Government-mandated lockdowns and social distancing measures significantly altered health behaviors—including smoking, alcohol consumption, dietary habits, and anxiety levels—and demonstrated the complex interrelationships between PA, behavioral changes, and mental well-being [9,10,11,12,13,14,15]. However, the relationship between PA, behavioral changes, and mental health during the pandemic remains unclear, with inconsistent findings across different populations. For example, while a UK study found higher levels of muscle-strengthening activity linked to increased alcohol consumption during the pandemic [16], an international survey reported a significant decline in binge drinking during COVID-19 confinement [17]. Similarly, studies on smoking behavior produced mixed findings: research in China reported a negative association between PA and smoking [18], whereas global data suggested that the pandemic acted as both a barrier and a facilitator for smoking cessation [12]. Eating behavior changes were also varied, ranging from stress-driven binge eating or dietary restriction [19] to increased emotional eating of processed foods, sweets, and alcohol [14,20]. Preventive health behaviors further highlighted these complexities—regular PA was associated with higher adherence to public health measures such as mask-wearing and handwashing in Mexico [21], yet mask mandates discouraged some individuals from engaging in PA, with 41% reporting reduced activity due to negative experiences with mask use [22]. Mental health findings were similarly mixed; while studies in China linked regular PA to lower anxiety and depression risks [23], global research indicated increased sedentary behavior and poorer psychological outcomes in some populations [24]. These inconsistencies emphasize the need for more targeted research to clarify PA’s role in behavioral and mental health adaptations during pandemics.

Despite valuable insights from previous studies, key research gaps remain. Most studies focus on younger populations [4,19] or rely on localized datasets [4,20,21,23], leaving a critical gap in understanding PA’s role among older adults in non-Western settings. Moreover, limited research examines how different domains of PA—such as leisure, occupational, or variations in intensity (light, moderate, vigorous)—affect health behaviors and mental well-being during a pandemic. Addressing these gaps is essential for developing targeted interventions for at-risk populations.

This study leverages data from the 2020 China Health and Retirement Longitudinal Study (CHARLS), collected shortly after the initial Wuhan lockdown [25], to investigate how various types and intensities of PA influenced health behaviors and mental well-being among middle-aged and older Chinese adults. Given the conflicting evidence in prior research, we hypothesize that engagement in PA is associated with changes in health behaviors (smoking, alcohol consumption, and dietary habits) and mental well-being (anxiety and fear) during the COVID-19 pandemic. Specifically, we examine the associations between each PA domain (categorized by intensity level and activity purpose) and these health-related outcomes in separate analyses. By utilizing data from the early stage of the COVID-19 pandemic, this study provides new insights into PA’s role in mitigating pandemic-related health impacts and offers evidence-based strategies to promote healthier behaviors during future public health crises.

## 2. Materials and Methods

### 2.1. Data Source and Study Population

This cross-sectional study utilizes the Chinese national data from wave 5 (2020) of the China Health and Retirement Longitudinal Study (CHARLS). CHARLS is a longitudinal study designed to represent individuals aged 45 and above residing in mainland China. The national baseline survey was conducted in 2011–2012, followed by subsequent waves in 2013 (wave 2), 2015 (wave 3), 2018 (wave 4), and 2020 (wave 5). Wave 5 was officially released on 16 November 2023 [25]. The baseline survey encompassed 28 provinces, 150 counties/districts, and 450 villages/urban communities across China, to ensure national and regional representation of the country’s middle-aged and older population [26]. CHARLS employed a stratified, multi-stage, probability proportional to size (PPS) random sampling approach, which stratified by region, urban vs. rural status, and GDP per capita. The baseline sample was weighted to closely match China’s 2010 Population Census, confirming its national representativeness. However, due to COVID-19 restrictions, three sampled villages in Xinjiang and some in Inner Mongolia could not be surveyed in wave 5, potentially leading to minor underrepresentation in those regions.

From late 2019 through early 2020, a COVID-19 outbreak occurred in China, and COVID-19-related data were collected in wave 5, documenting the pandemic’s effects on the lives and health of middle-aged and older Chinese adults [25]. Ethical approval for all waves of CHARLS was granted by the Institutional Review Board (IRB) at Peking University. The IRB approval number for the main household survey is IRB00001052-11015.

**Figure 1:**
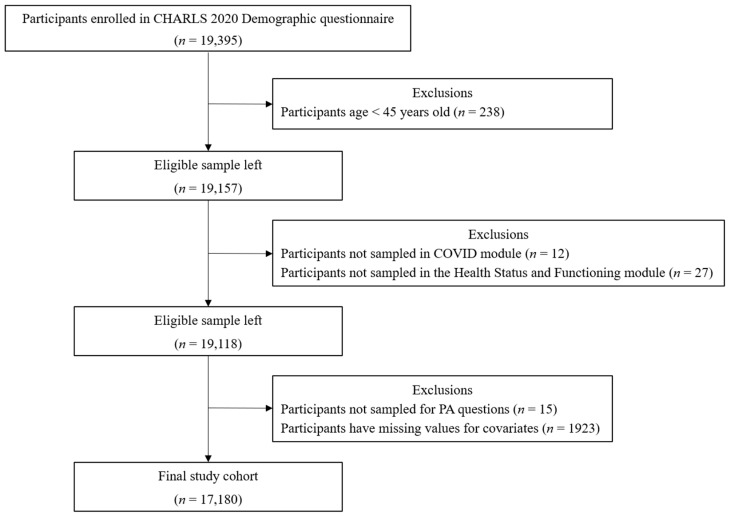
Flowchart of data selection and exclusion procedure.

The 2020 CHARLS dataset contained demographic information for 19,395 individuals. Exclusions were made based on multiple criteria: a total of 238 individuals were below the age of 45; 12 individuals were not included in the COVID questionnaire; 27 individuals were not sampled for the Health Status and Functioning questionnaire; 15 individuals had not participated in the PA questions; and 1923 individuals had incomplete covariate data. Following the removal of these exclusions, a total of 17,180 individuals remained in the final study cohort for further analysis. Listwise deletion was used to handle missing data, ensuring a complete dataset for all key study variables. The detailed procedure of selection is shown in Figure 1.

### 2.2. Measures

#### 2.2.1. Measurement of Behavioral Changes and Mental Health

Drawing from previous research [10,27,28], we identified relevant dependent variables in the Module V COVID-19 section of CHARLS 2020 [29] that pertained to health behavior changes, mental health status, and panic purchasing behaviors driven by negative emotions such as fear, sadness, and anxiety. Health behavior changes, including smoking, alcohol consumption, and diet habits, were classified as increased (increased slightly or greatly) and decreased (decreased slightly or greatly) in comparison to the prior time had the pandemic not happened. Individuals who were abstinent from alcohol or non-smokers in recent years were excluded from the analysis, as the focus of this study was on changes in consumption behaviors among those who reported alcohol or tobacco use during the pandemic. Mask-wearing during the pandemic was classified as affirmative (always or sometimes) and negative (never). Anxiety and fear during the coronavirus outbreak were assessed based on self-reported perceptions rather than standardized psychological instruments. Participants responded to the questions: “Have you ever felt stressful or anxious about the pandemic or anything related to the pandemic?” and “Have you ever felt the fear of the pandemic, or any fear related to the pandemic?” Responses were categorized into two groups: experiencing anxiety/fear (not often, sometimes, or often) and devoid of anxiety/fear (rarely or never). Panic purchasing was assessed through the question, “During the three days after Wuhan was closed due to the pandemic, that is, from the Chinese New Year’s Eve to the second day of the first Lunar Month (from January 24 to January 26), did you buy more of the following things ((1) food, cooking oil, and vegetables; (2) face masks, hand sanitizer, and disinfectant) than usual to stock up because of the pandemic?” Panic purchasing of food, and panic purchasing of personal protective equipment (PPE) were coded as either “yes” or “no” based on the respondent’s choices on this multiple-choice question.

#### 2.2.2. Assessment of Physical Activity

The CHARLS questionnaire [29] asked respondents whether they perform for at least 10 min continuously for a usual week in three different levels of PA: vigorous physical activity (VPA), which includes activities such as carrying heavy objects, digging, hoeing, aerobic workouts, bicycling at a fast speed, riding a cargo bike/motorcycle, etc.; moderate physical activity (MPA), which includes activities such as carrying light objects, bicycling at a normal speed, mopping, Tai-Chi, and speed walking; and light physical activity (LPA), which includes activities such as walking from one place to another at a workplace or home or taking walks for leisure, sports, exercise, or entertainment. Subsequent questions were documented on the days (1–7 d), time duration (10–30 min, 30 min–2 h, 2–4 h, and >4 h), and purpose (job demand, entertainment, exercise, or others) of engaging in such a type of PA during a usual week. This section of the questionnaire followed the format and statements of the short version of the International Physical Activity Questionnaire (IPAQ) [30]. VPA, MPA, and LPA were defined based on participants engaging in the respective activities for at least 10 consecutive minutes during a typical week. Participants who did not meet this criterion were classified as physically inactive.

To thoroughly evaluate the health benefits and assess various positive health implications of PA, three indicators were established that emphasize sufficient PA as a protective factor. Total physical activity (TPA) measures total PA across all domains; leisure physical activity (LePA) evaluates for entertainment and exercise; and occupational physical activity (OPA) exclusively addresses work-related activity. For the calculations, the duration of each level of PA was estimated using the median time range for each group, with durations exceeding 4 h being capped at 4 h. The total physical activity volume (TPAV), leisure physical activity volume (LePAV), and occupational physical activity volume (OPAV) scores were calculated by multiplying the weighted PAs using the metabolic equivalent (MET) [31,32,33] as follows: (1) TPAV = 8.0 × total VPA weekly duration score + 4.0 × total MPA weekly duration score + 3.3 × total LPA weekly duration score; (2) LePAV = 8.0 × leisure VPA weekly duration score + 4.0 × leisure MPA weekly duration score + 3.3 × leisure LPA weekly duration score; (3) OPAV = 8.0 × occupational VPA weekly duration score + 4.0 × occupational MPA weekly duration score + 3.3 × occupational LPA weekly duration score. Following the IPAQ guidelines, TPA, LePA, and OPA were defined as reaching at least 600 MET-min per week for TPAV, LePAV, and OPAV, respectively. Participants falling short of this threshold were categorized as physically inactive.

Covariates were selected based on previous research [16,33,34,35,36] and the availability of data from the 2020 CHARLS. These included: (1) sociodemographic factors such as age (divided into two categories by the median), gender (male or female), education level (less than lower secondary education, upper secondary and vocational training, and tertiary education), marital status (married or separated/no longer living with a spouse), and residence (urban or rural); and (2) comorbidities and health status, including chronic disease presence (having at least one chronic disease or none) and self-reported health status (good or poor). The definitions and assignments of variables are presented in Table 1.

### 2.3. Statistical Analysis

The characteristics of the overall study population and each PA group were presented as counts and percentages. Chi-squared tests were used to assess the association between PA groups and the various characteristic variables.

Given the hierarchical structure of the CHARLS data, where individuals (Level 1) are nested within households (Level 2), and households within communities (Level 3), a generalized linear mixed-effects model (GLMM) with random intercepts was employed. GLMMs [37] are well-suited for analyzing multilevel data, accounting for correlations at multiple levels while modeling individual-level relationships between predictors and outcomes. By incorporating random effects at the household and community levels, they control for within-group dependencies and improve the accuracy of fixed-effect estimations. Compared to other statistical models, GLMMs naturally accommodate a hierarchical data structure, ensuring more precise inferences regarding the associations between PA, health behaviors, and mental well-being in a nested dataset like CHARLS.

The GLMM estimated odds ratio (OR), 95% confidence interval (95% CI), and *p*-value of the fixed effects using maximum likelihood methods with bound optimization by quadratic approximation. The regression model is presented in Equation (1); each model was performed independently, with one outcome and one exposure included at a time:

(1) $$g\left[E\left(y_{ijk}|u_{i},\;u_{j\left(i\right)}\right)\right]=X_{ijk}\beta +u_{i}+u_{j\left(i\right)}+e_{ijk}$$

where $\;y_{ijk}\;$ is the response of the ${\;k}^{th}\;$ individual in the ${\;j}^{th}\;$ household-level unit of the ${\;i}^{th}\;$ community unit. The matrix $X_{ijk}\;$ is the design matrix associated with the fixed effect, and  β  represents unknown regression coefficients. The term of ${\;u}_{i}\;$ is the random intercept effect for community-level variability, distributed as $\;N\left(0,\;\sigma_{c}^{2}\right)$; the term of ${\;u}_{j\left(i\right)}\;$ is the random intercept effect for household-level variability, distributed as $\;N(0,\;\sigma_{h}^{2})$. The random error term, $\;e_{ijk}$, is distributed as $\;N(0,\;\sigma_{e}^{2})$. For a binary outcome, a logistic link function is used, where $\;g\left(\cdot \right)={log}_{e}(\frac{p}{1-p})$.

All *p*-values were calculated using two-sided tests and considered statistically significant at the 0.05 test level. All statistical analyses were conducted using R (cran.r-project.org) version 4.4.1 and SAS (SAS Inst. Inc., Cary, NC, USA) version 9.4.

## 3. Results

### 3.1. Descriptive Statistic

Table 2 displays the detailed distribution of PA within the study cohort, along with their associations with the characteristics of all covariates. Among the participants, 6389 engaged in VPA, 9946 in MPA, and 13,422 in LPA. Additionally, 14,599 participants were categorized as TPA, 7838 as LePA, and 7539 as OPA. While younger participants were more likely to engage in VPA and MPA but also more likely to experience physical inactivity related to leisure, older adults were more likely to engage in LPA and to experience both occupational and total physical inactivity. Although females were less likely to be reported in TPA, LePA, and OPA, they tended to be engaged more in MPA. In terms of sociodemographic and health status, participants who engaged in TPA, LePA, and OPA were more likely to be married, have a low level of education, live in a rural region, be free from chronic diseases, have a positive perception of their own health, and be without anxiety. More information on the statistical characteristics of the participants is shown in Table 2.

### 3.2. Association Between Physical Activity and Health Behavioral Changes Among Chinese Middle-Aged and Older Adults

Table 3 and Table 4 present the estimated odds ratios for the associations between PA domains and health behavior changes during the pandemic, with Table 3 showing the unadjusted results and Table 4 reflecting the adjusted models.

In the unadjusted models, LePA and OPA showed opposite associations with smoking and drinking behaviors. Individuals engaged in LePA had 29% lower odds of increased smoking (OR = 0.71, 95% CI: 0.57–0.90) and 30% lower odds of increased drinking (OR = 0.70, 95% CI: 0.54–0.90), suggesting that LePA may serve as a protective factor for reducing these behaviors. Conversely, individuals engaged in OPA had 45% higher odds of increased smoking (OR = 1.45, 95% CI: 1.15–1.83) and 43% higher odds of increased alcohol consumption (OR = 1.43, 95% CI: 1.10–1.86). Although both findings were statistically significant, the stronger association with OPA highlights a more substantial public health concern, particularly in the context of work-related stressors during the pandemic. Additionally, VPA was associated with 43% higher odds of increased alcohol consumption (OR = 1.43, 95% CI: 1.11–1.85), while MPA and LePA were associated with 33% and 29% higher odds of increased food intake, respectively (MPA: OR = 1.33, 95% CI: 1.08–1.65; LePA: OR = 1.29, 95% CI: 1.05–1.58). These results suggest that PA, particularly LePA and MPA, may influence dietary habits in response to stress or lifestyle adjustments during the pandemic. Furthermore, all PA domains were significantly associated with higher odds of mask-wearing and panic purchasing of food and PPE, highlighting the potential role of PA in reinforcing health-conscious behaviors during public health crises.

After adjusting for potential confounders—including age, gender, education level, marital status, residency, chronic disease status, and self-reported health—the associations between PA and health behaviors remained largely consistent. However, some associations weakened. Specifically, some odds ratios of OPA were slightly attenuated, rendering them no longer statistically significant and suggesting that other factors may explain this behavior. Notably, LePA remained significantly associated with 23% lower odds of increased smoking (OR = 0.77, 95% CI: 0.60–0.98)**,** reinforcing the idea that leisure-based PA could serve as a protective factor against unhealthy behaviors such as smoking. Similarly, VPA remained significantly associated with 42% higher odds of alcohol consumption (OR = 1.42, 95% CI: 1.08–1.86), suggesting that individuals engaging in more intense PA might also be prone to increased alcohol use, potentially as a coping mechanism for stress.

Overall, these findings suggest that PA engagement during the pandemic was associated with shifts in health behaviors, with distinct patterns observed across different PA domains. LePA was generally associated with health-protective behaviors, while OPA was linked to riskier behaviors such as increased smoking and drinking.

### 3.3. Association Between Physical Activity and Mental Health Changes Among Chinese Middle-Aged and Older Adults

Table 3 and Table 4 also present the associations between PA engagement and mental health outcomes (anxiety and fear) during the pandemic.

In the unadjusted models, individuals who engaged in all PA domains had significantly higher odds of experiencing anxiety and fear compared to physically inactive individuals, except for LePA, which showed a slight but non-significant association with fear. Notably, individuals who engaged in MPA had 50% higher odds of experiencing anxiety (OR = 1.50, 95% CI: 1.40–1.61) and 58% higher odds of experiencing fear (OR = 1.58, 95% CI: 1.47–1.69), suggesting that moderate levels of PA might have heightened psychological distress during the pandemic.

After adjusting for covariates, the findings remained consistent: individuals engaging in any domain of PA had significantly higher odds of experiencing anxiety and fear compared to those who were inactive. The associations were particularly pronounced for individuals engaged in VPA (Anxiety: OR = 1.31, 95% CI: 1.22–1.42; Fear: OR = 1.30, 95% CI: 1.20–1.40), MPA (Anxiety: OR = 1.33, 95% CI: 1.24–1.42; Fear: OR = 1.38, 95% CI: 1.28–1.48), and TPA (Anxiety: OR = 1.42, 95% CI: 1.28–1.57; Fear: OR = 1.49, 95% CI: 1.35–1.65). These results suggest that while PA is generally associated with better mental health in normal circumstances, the context of a public health crisis, particularly with lockdowns and uncertainty, may have exacerbated pandemic-related fears and anxieties among active individuals.

Overall, these results indicate that PA engagement was paradoxically associated with heightened anxiety and fear during the pandemic, emphasizing the need for mental health interventions tailored to active individuals during public health crises.

## 4. Discussion

Using 2020 data from CHARLS, we identified associations between usual-week physical activities and changes in health behaviors, as well as a positive association between usual-week PA and the experience of anxiety and fear during the pandemic, which subsequently influenced the propensity for panic purchases of food and PPE. This study also found that LePA and OPA have opposite impacts on behavior changes in smoking, drinking, and eating during the pandemic outbreak. To the best of our knowledge, this is the first study to explore the association between six domains of regular PA and health behavior changes, and the relationship between regular PA and mental status, among Chinese middle-aged and older adults during the pandemic.

This study provided a unique perspective into associations between mental status/behaviors driven by mental status and all six domains of PA. Specifically, middle-aged and older adults who engaged in regular weekly PA were positively associated with anxiety and fear during the pandemic outbreak in China. While this finding contradicts prior studies suggesting that PA generally reduces stress and anxiety [7,38,39,40], it aligns with more recent research that highlights the complex psychological responses to pandemic-related disruptions [41,42]. Moreover, it is important to acknowledge that individual experiences of anxiety and fear may vary across demographic subgroups such as age, gender, and socioeconomic status. For example, older adults may have been more concerned about COVID-19’s health risks, while younger adults may have faced heightened financial uncertainty. Gender differences in caregiving responsibilities and employment disruptions may also have influenced mental health outcomes. Future research should investigate these subgroup differences to better understand how PA-related psychological responses differ based on demographic characteristics.

One possible explanation for this paradoxical association lies in the Health Belief Model (HBM) [43], which posits that individuals’ health behaviors are influenced by perceived susceptibility, severity, benefits, and barriers. Active individuals tend to be more health-conscious and may have perceived themselves as highly vulnerable to disruptions in their routines due to lockdown measures. Given their heightened awareness of the importance of maintaining an active lifestyle for both physical and mental well-being, they may have experienced greater anxiety regarding restrictions on movement, gym closures, and limited access to outdoor activities. This heightened perceived susceptibility to the adverse effects of inactivity could have contributed to increased psychological distress during the pandemic. Furthermore, the Allostatic Load Theory (ALT) [44] provides another theoretical explanation. This theory describes how chronic exposure to stressors—including public health crises like COVID-19—leads to cumulative physiological wear and tear, ultimately affecting mental health outcomes. During the pandemic, individuals with habitual PA routines may have faced significant lifestyle disruptions, leading to increased stress levels due to concerns about maintaining their fitness, immune function, and long-term health. This heightened stress response, compounded by pandemic-related uncertainties, may have contributed to the observed associations between higher PA engagement and increased fear and anxiety. Additionally, the timing of data collection may have played a role in these findings. The CHARLS questionnaire assessing anxiety and fear was administered during the critical outbreak period (25 January to 22 February 2020 [25]), when lockdowns [45], travel restrictions [46], and shortages of protective equipment were at their peak in China [47,48]. Individuals with regular exercise habits might have felt particularly vulnerable to these disruptions, intensifying their stress responses. Moreover, differences in access to outdoor spaces, financial stability, and health literacy may have further shaped individual responses to the pandemic. Those with fewer resources or limited ability to engage in LePA may have experienced greater stress due to disruptions in their routines and heightened concerns about health and economic stability. Therefore, due to the heightened fears that the outbreak of COVID-19 might lead to changes in health behaviors such as decreased PA [49], individuals who regularly engage in PA may be more likely to adopt preventive health behaviors, such as stockpiling food, purchasing protective equipment, and closely monitoring health-related information, which could further reinforce pandemic-related anxiety.

In the first three days after Wuhan’s lockdown due to the pandemic, our study identified no significant association between occupational PA and behaviors such as panic purchases of food and PPE. This may be attributed to the specific nature of work-related PA, which differs from other domains of PA, such as leisure or household activities, which tend to be more adaptable and closely integrated with personal routines. Additionally, OPA typically refers to the movement within one’s professional role. Individuals engaged in OPA may have prioritized concerns regarding anxiety and fears related to job security or the transition to remote work over immediate health threats. This assumption was further supported by the insignificant association between OPA and wearing a mask during the pandemic. Apart from OPA and VPA, participants who engaged in MPA, LPA, TPA, and LePA exhibited a significant positive association with mask-wearing. This aligned with the existing literature [50,51,52], suggesting that individuals who regularly engage in PA tend to possess heightened health consciousness and are more inclined to stay informed about public health guidelines. These individuals recognize the significance of preventive measures like mask-wearing, particularly in settings with elevated risks of COVID-19 transmission. However, the negative association between VPA and mask-wearing may be attributed to several factors, including the discomfort and breathing difficulties caused by wearing a mask, and a lower risk of transmission in outdoor environments [53].

In line with other studies [16,54], the results showed that higher rates of intensive PA significantly increased alcohol consumption during the pandemic outbreak. High-intensity workouts are known to reduce stress levels, but some individuals may also turn to alcohol as a coping mechanism for relaxation or escape when social activities are limited [55]. Participants who engaged in LePA significantly reduced alcohol use, decreased smoking, and increased food intake, which confirmed that participants who consistently engaged in regular leisure-time PA are more likely to adopt other health-conscious behaviors to improve their overall well-being and avoid health risks during the pandemic, such as avoiding harmful habits like smoking and drinking and ensuring a balanced diet [56,57]. However, OPA exhibited a contrasting pattern, with participants engaged in OPA showing increased smoking, alcohol use, and reduced food intake, highlighting adverse health behavior changes relative to LePA. This finding aligns with the perspective that individuals engaged in OPA experienced heightened anxiety regarding job uncertainty during the outbreak, rather than concerns about their health. The socio-cultural context of China may further explain these observed associations between OPA and health behavior changes. Unlike structured workplace environments in Western countries, OPA in China, especially in rural areas, is often characterized by labor-intensive agricultural and manual work, which continued despite pandemic restrictions [58]. Many individuals engaged in OPA faced economic pressures and job insecurity, as their work was essential but lacked the flexibility of remote employment. The necessity to maintain livelihoods may have led to increased stress levels, influencing behaviors such as smoking, alcohol consumption, and dietary habits. Additionally, China’s strong collectivist culture and intergenerational family structures [59] may have influenced PA engagement and health behavior changes during the pandemic. Middle-aged and older adults often prioritize their family’s well-being over their own health. Balancing work, caregiving, and household responsibilities may limit their ability to engage in structured PA. Unlike in individualistic societies where PA is often pursued for personal health benefits, PA engagement in China is deeply intertwined with familial obligations and social structures, further shaping its impact on health behaviors.

Moreover, the blurred boundaries between work and daily life in rural and informal employment settings may have contributed to these contrasting effects of OPA and LePA. Unlike LePA, which is a conscious health-promoting activity, OPA may not provide the same psychological or physiological benefits, reinforcing divergent health behavior patterns. Compared to individuals with sedentary jobs or those working from home, OPA workers may have been more affected by the disruptions caused by the pandemic. This could explain why those engaged in OPA exhibited unfavorable changes in health behaviors, as they navigated heightened financial uncertainty and workplace instability. Lastly, no significant association was identified between any categories of PA and changes in sleep, suggesting that PA intensity alone may not directly impact sleep behaviors in the context of a public health crisis.

The present study has some limitations. First, PA, health behavior changes, and mental health outcomes were all self-reported, which may introduce potential biases, such as recall bias and social desirability bias. These limitations suggest that the observed associations between PA and health behaviors, while valuable, should be interpreted with caution. In particular, anxiety and fear were assessed based on respondents’ self-perceived frequency rather than standardized scientific instruments, such as the Center for Epidemiologic Studies Depression Scale (CES-D-10). Specifically, these outcomes were evaluated by frequency categories (rarely or never, not often, sometimes, often) rather than using validated scales. While this approach was constrained by data availability, the use of standardized measures would have enhanced the reliability and comparability of the findings. Moreover, there was no clear classification of mental conditions based on their underlying causes. Additionally, pandemic-specific stress factors, individual coping strategies, and potential regional disparities in health behaviors were not examined due to data limitations, leaving a gap in understanding the potential effects of PA on mental health during such crises. Future studies should address these aspects to provide a more comprehensive analysis.

Second, listwise deletion was applied to handle missing data, ensuring a complete dataset for analysis. While this approach minimizes bias in estimation, it may introduce some selection bias, as individuals with incomplete covariate data were excluded from the final analytical sample. Future research may consider multiple imputation techniques to assess the robustness of findings when handling missing data. Additionally, we excluded the “no change” group from the analysis of changes in smoking, drinking, and eating behaviors due to the high percentage of participants in this category. This exclusion was necessary to maintain statistical validity, as including the “no change” group would have substantially influenced regression estimates and could not be combined with either the “increased” or “decreased” group. As a result, the study sample used to examine PA’s associations with changes in drinking, smoking, and eating behaviors was smaller than in other analyses, potentially introducing bias and limiting the generalizability of these specific findings. Third, BMI and other anthropometry variables, such as height, weight, and waist circumference data, were not available in CHARLS since wave 4 (2018). The absence of these variables may reduce the model’s validity, particularly in assessing PA’s relationship with overall health. Fourth, due to space and data constraints, we did not further classify TPA, LePA, and OPA into vigorous, moderate, and light intensity categories. This classification could provide deeper insights into how different PA intensities influence health behaviors. Similarly, potential interactions between different PA types were not examined in this study. Future research should investigate whether various PA domains interact in shaping health behavior modifications and mental health outcomes, as such interactions may provide a more nuanced understanding of PA’s role in behavioral adaptations during public health crises. Finally, while our study focused on immediate psychological responses during the pandemic, it is critical to consider potential long-term mental health consequences. Although heightened anxiety during the pandemic may have been an acute response to external stressors, prolonged psychological distress could contribute to chronic mental health issues, including sleep disturbances and maladaptive coping mechanisms such as increased alcohol consumption or social avoidance. Longitudinal studies are needed to examine whether these psychological effects persisted beyond the acute phase of the pandemic and how they may have influenced future PA behaviors and overall mental health trajectories.

## 5. Conclusions

This study comprehensively examines the associations between six domains of PA and health behavior changes, as well as their relationships with mental well-being, during the COVID-19 outbreak among middle-aged and older adults in China. Our findings support the hypothesis that PA engagement is associated with changes in smoking, alcohol consumption, dietary habits, anxiety, and fear during the pandemic. Specifically, LePA was positively associated with increased anxiety, fear, and panic purchasing behaviors, while also leading to beneficial changes such as reduced smoking and alcohol consumption and increased food intake. This suggests that while individuals engaging in leisure-time PA may have adopted healthier behaviors, they also exhibited heightened psychological distress due to pandemic-related disruptions. Conversely, OPA exhibited opposite associations with these behaviors, being linked to increased smoking and alcohol consumption, which may reflect the influence of occupational stress and job-related uncertainties during the pandemic. These findings highlight the distinct effects of PA domains on behavioral adaptations during public health crises.

In light of these findings, the following recommendations are proposed: First, developing mental health interventions specifically tailored to managing anxiety and fear during pandemics is crucial. These interventions could include community-based mental health programs, telehealth counseling services, and education campaigns focused on coping strategies to reduce stress and panic purchasing behaviors. Second, it is essential to separate PA into leisure-related and work-related activities due to their distinct impacts on health behaviors, well-being, and lifestyle. Public health initiatives could promote leisure-time PA through accessible home-based exercise programs, while addressing workplace stressors to mitigate negative effects associated with occupational PA. Finally, while this study did not find significant effects of varying PA levels on some health behavior changes, future longitudinal research is necessary to further investigate these associations. Such research should examine how different intensities and domains of PA influence long-term health behavior changes, particularly under crisis conditions like pandemics.

## Figures and Tables

**Table 1:** Summary of variable definitions and assignments.

Variables	Question Number Labeled in CHARLS’ Codebook	Definition	Assignment
Dependent Variables			
Change in Smoking	vc014	The amount of smoking increased or decreased during the Lunar New Year outbreak compared to what it would have been if the pandemic had not happened.	Decreased slightly/greatly = 0, Increased slightly/greatly = 1
Change in Drinking	vc015	The amount of alcohol consumed increased or decreased during the Lunar New Year outbreak compared to what it would have been if the pandemic had not happened.	Decreased slightly/greatly = 0, Increased slightly/greatly = 1
Change in Eating	vc017	The amount of food eaten each day increased or decreased during the Lunar New Year outbreak compared to what it would have been if the pandemic had not happened.	Decreased slightly/greatly = 0, Increased slightly/greatly = 1
Mask-Wearing	va003	Masking during the pandemic.	Never = 0, Always/Sometimes = 1
Anxiety	vc013	Feeling anxiety about the pandemic or anything related to the pandemic during the outbreak.	Rarely or never = 0, Not often/sometimes/often = 1
Fear	vc012	Feeling fears about the pandemic or anything related to the pandemic during the outbreak.	Rarely or never = 0, Not often/sometimes/often = 1
Panic Purchasing of Food	va006_s1	Had to buy more face masks, hand sanitizer and disinfectant than usual to stock up because of the pandemic during the three days after Wuhan was closed due to the pandemic (from January 24 to January 26).	No = 0, Yes = 1
Panic Purchasing of Personal Protective Equipment	va006_s2	Had to buy more food, cooking oil, and vegetables than usual to stock up because of the pandemic during the three days after Wuhan was closed due to the pandemic (from January 24 to January 26).	No = 0, Yes = 1
Independent Variables			
Vigorous Physical Activity (VPA)	da032_1_	Performing vigorous physical activity for at least 10 min at a time during a usual week.	No = 0, Yes = 1
Moderate Physical Activity (MPA)	da032_2_	Performing moderate physical activity for at least 10 min at a time during a usual week.	No = 0, Yes = 1
Light Physical Activity (LPA)	da032_3_	Performing light physical activity for at least 10 min at a time during a usual week.	No = 0, Yes = 1
Total Physical Activity (TPA)	da032–da036	The total physical activity volume reached at least 600 MET-min per week.	No = 0, Yes = 1
Leisure Physical Activity (LePA)	da032–da037	The leisure physical activity volume (for the purpose of entertainment and exercise) reached at least 600 MET-min per week.	No = 0, Yes = 1
Occupational Physical Activity (OPA)	da032–da037	The occupational physical activity volume (for the purpose of job demands) reached at least 600 MET-min per week.	No = 0, Yes = 1
Covariates			
Age	xrage	Age per the solar calendar at the time of interview.	<61 y/o = 0, ≥61 y/o = 1
Gender	xrgender	Biological sex	Male = 0, Female = 1
Education Level	ba010	Highest level of education.	less than lower secondary education = 0, upper secondary and vocational training = 1, tertiary education = 2
Marital Status	ba011	Married, included both married and temporarily living apart from their spouse due to circumstances like a job; the category of not married includes those who are separated or no longer living together as a spouse, divorced, widowed, or never married.	Not married = 0, Married = 1
Residency	ba008	Urban, included city center or town center; combined zone included area between urban and rural areas, or Zhenxiang area; the village area was rural.	Rural = 0, Combined Zone =1, Urban = 2
Chronic Diseases	da003_1_–da003_15_	Has the doctor diagnosed the respondent with any of the following chronic diseases: hypertension, dyslipidemia, diabetes, or high blood sugar, cancer or malignant tumor, chronic lung diseases, liver disease, heart disease, stroke, kidney disease, stomach or other digestive diseases, emotional, nervous, or psychiatric problems, memory-related disease, Parkinson’s disease, arthritis or rheumatism, and asthma (multiple choices allowed).	None = 0, At least has any one type of chronic diseases = 1
Self-Reported Health	da001	Self-assessment of one’s own health status.	Very poor/poor/fair = 0, Very good/good = 1

**Table 2:** Characteristics of participants in study cohort.

Variables		Vigorous Physical Activity (VPA)			Moderate Physical Activity (MPA)			Light Physical Activity (LPA)			Total Physical Activity (TPA)			Leisure Physical Activity (LPA)			Occupational Physical Activity (OPA)		
		No	Yes	*p* Value	No	Yes	*p* Value	No	Yes	*p* Value	No	Yes	*p* Value	No	Yes	*p* Value	No	Yes	*p* Value
*N*		10,791	6389		7234	9946		3758	13,422		2621	14,599		9342	7838		9641	7539	
Age, *n* (%)	<61 y/o	4751 (44.03)	3599 (56.33)	<0.001	2952 (40.81)	5398 (54.27)	<0.001	1692 (45.02)	6658 (49.61)	<0.001	971 (37.05)	7379 (50.68)	<0.001	4951 (53.00)	3399 (43.37)	<0.001	3785 (39.26)	4565 (60.55)	<0.001
	≥61 y/o	6040 (55.97)	2790 (43.67)		4282 (59.19)	4548 (45.73)		2066 (54.98)	6764 (50.39)		1650 (62.95)	7180 (49.32)		4391 (47.00)	4439 (56.63)		5856 (60.74)	2974 (39.45)	
Gender, *n* (%)	Male	4727 (43.81)	3353 (52.48)	<0.001	3943 (54.51)	4137 (41.59)	<0.001	1724 (45.88)	6356 (47.36)	0.108	1186 (45.25)	6894 (47.35)	0.047	4439 (47.52)	3641 (46.45)	0.164	4150 (43.05)	3930 (52.13)	<0.001
	Female	6064 (56.19)	3036 (47.52)		3291 (45.49)	5809 (58.41)		2034 (54.12)	7066 (52.64)		1435 (54.75)	7665 (52.65)		4903 (52.48)	4197 (53.55)		5491 (56.95)	3609 (47.87)	
Education Level, *n* (%)	Less than lower secondary education	9159 (84.88)	5802 (90.81)	<0.001	6416 (88.69)	8545 (85.91)	<0.001	3487 (92.79)	11,474 (85.49)	<0.001	2399 (91.53)	12,562 (86.28)	<0.001	8462 (90.58)	6499 (82.92)	<0.001	8147 (84.50)	6814 (90.38)	<0.001
	Upper secondary and vocational training	1341 (12.43)	506 (7.92)		698 (9.65)	1149 (11.55)		242 (6.44)	1605 (11.96)		193 (7.36)	1654 (11.36)		774 (8.29)	1073 (13.69)		1197 (12.42)	650 (8.62)	
	Tertiary education	291 (2.70)	81 (1.27)		120 (1.66)	252 (2.53)		29 (0.77)	343 (2.56)		29 (1.11)	343 (2.36)		106 (1.13)	266 (3.39)		297 (3.08)	75 (0.99)	
Marital Status, *n* (%)	Not married	1898 (17.59)	676 (10.58)	<0.001	1301 (17.98)	1273 (12.80)	<0.001	641 (17.06)	1933 (14.40)	<0.001	581 (22.17)	1993 (13.69)	<0.001	1267 (13.56)	1307 (16.68)	<0.001	1881 (19.51)	693 (9.19)	<0.001
	Married	8893 (82.41)	5713 (89.42)		5933 (82.02)	8673 (87.20)		3117 (82.94)	11,489 (85.60)		2040 (77.83)	12,566 (86.31)		8075 (86.44)	6531 (83.32)		7760 (80.49)	6846 (90.81)	
Residency, *n* (%)	Rural	6050 (56.07)	4985 (78.02)	<0.001	4750 (65.66)	6285 (63.19)	0.003	2779 (73.95)	8256 (61.51)	<0.001	1808 (68.98)	9227 (63.38)	<0.001	6717 (71.90)	4318 (55.09)	<0.001	5417 (56.19)	5618 (74.52)	<0.001
	Combined zone	1448 (13.42)	648 (10.14)		862 (11.92)	1234 (12.41)		428 (11.39)	1668 (12.43)		312 (11.90)	1784 (12.25)		1033 (11.06)	1063 (13.56)		1252 (12.99)	844 (11.20)	
	Urban	3293 (30.52)	756 (11.83)		1622 (22.42)	2427 (24.40)		551 (14.66)	3498 (26.06)		501 (19.11)	3548 (24.37)		1592 (17.04)	2457 (31.35)		2972 (30.83)	1077 (14.29)	
Chronic Diseases, *n* (%)	None	6745 (62.51)	4093 (64.06)	0.041	4554 (62.95)	6284 (63.18)	0.759	2397 (63.78)	8441 (62.89)	0.315	1634 (62.34)	9204 (63.22)	0.392	6008 (64.31)	4830 (61.62)	<0.001	5932 (61.53)	4906 (65.07)	<0.001
	At least has any one type of chronic diseases	4046 (37.49)	2296 (35.94)		2680 (37.05)	3662 (36.82)		1361 (36.22)	4981 (37.11)		987 (37.66)	5355 (36.78)		3334 (35.69)	3008 (38.38)		3709 (38.47)	2633 (34.93)	
Self-Reported Health, *n* (%)	Poor	8190 (75.90)	4751 (74.36)	0.024	5459 (75.46)	7482 (75.23)	0.722	2891 (76.93)	10,050 (74.88)	0.001	2054 (78.37)	10,887 (74.78)	<0.001	7122 (76.24)	5819 (74.24)	0.003	7369 (76.43)	5572 (73.91)	<0.001
	Good	2601 (24.10)	1638 (25.64)		1775 (24.52)	2464 (24.77)		867 (23.07)	3372 (25.12)		567 (21.63)	3672 (25.22)		2220 (23.76)	2019 (25.76)		2272 (23.57)	1967 (26.09)	
Anxiety, *n* (%)	No	6915 (64.08)	3724 (58.29)	<0.001	4819 (66.62)	5820 (58.52)	<0.001	2489 (66.23)	8150 (60.72)	<0.001	1777 (67.80)	8862 (60.87)	<0.001	5825 (62.35)	4814 (61.42)	0.209	6174 (64.04)	4465 (59.23)	<0.001
	Yes	3876 (35.92)	2665 (41.71)		2415 (33.38)	4126 (41.48)		1269 (33.77)	5272 (39.28)		844 (32.20)	5697 (39.13)		3517 (37.65)	3024 (38.58)		3467 (35.96)	3074 (40.77)	

**Table 3:** Results of the unadjusted model examining associations between PA domains and changes in health behaviors and mental health outcomes.

	Change in Smoking (*n* = 1426)		Change in Drinking (*n* = 1923)		Change in Eating (*n* = 2576)		Mask-Wearing (*n* = 16,197)		Anxiety (*n* = 17,180)		Fear (*n* = 17,160)		Panic Purchasing of Food (*n* = 17,180)		Panic Purchasing of Personal Protective Equipment (*n* = 17,180)	
	OR (95% CI)	*p* Value	OR (95% CI)	*p* Value	OR (95% CI)	*p* Value	OR (95% CI)	*p* Value	OR (95% CI)	*p* Value	OR (95% CI)	*p* Value	OR (95% CI)	*p* Value	OR (95% CI)	*p* Value
Vigorous Physical Activity (VPA)																
No	Ref		Ref		Ref		Ref		Ref		Ref		Ref		Ref	
Yes	1.22 (0.97, 1.53)	0.095	1.43 (1.11, 1.85)	0.006	0.83 (0.68, 1.03)	0.088	0.86 (0.77, 0.95)	0.002	1.28 (1.19, 1.37)	<0.001	1.27 (1.18, 1.36)	<0.001	1.14 (1.05, 1.24)	0.001	1.13 (1.02, 1.24)	0.015
Moderate Physical Activity (MPA)																
No	Ref		Ref		Ref		Ref		Ref		Ref		Ref		Ref	
Yes	1.17 (0.93, 1.46)	0.184	1.05 (0.81, 1.37)	0.691	1.33 (1.08, 1.65)	0.008	1.5 (1.37, 1.66)	<0.001	1.50 (1.40, 1.61)	<0.001	1.58 (1.47, 1.69)	<0.001	1.33 (1.23, 1.43)	<0.001	1.44 (1.31, 1.58)	<0.001
Light Physical Activity (LPA)																
No	Ref		Ref		Ref		Ref		Ref		Ref		Ref		Ref	
Yes	0.79 (0.6, 1.04)	0.088	0.97 (0.69, 1.36)	0.846	1.07 (0.83, 1.39)	0.588	1.33 (1.19, 1.49)	<0.001	1.31 (1.21, 1.43)	<0.001	1.36 (1.25, 1.48)	<0.001	1.34 (1.22, 1.47)	<0.001	1.24 (1.11, 1.39)	<0.001
Total Physical Activity (TPA)																
No	Ref		Ref		Ref		Ref		Ref		Ref		Ref		Ref	
Yes	1.26 (0.91, 1.74)	0.167	1.54 (0.97, 2.43)	0.066	1.32 (0.98, 1.80)	0.072	1.48 (1.30, 1.69)	<0.001	1.44 (1.31, 1.59)	<0.001	1.52 (1.38, 1.67)	<0.001	1.46 (1.32, 1.63)	<0.001	1.39 (1.21, 1.59)	<0.001
Leisure Physical Activity (LePA)																
No	Ref		Ref		Ref		Ref		Ref		Ref		Ref		Ref	
Yes	0.71 (0.57, 0.90)	0.005	0.70 (0.54, 0.90)	0.006	1.29 (1.05, 1.58)	0.013	1.20 (1.09, 1.32)	<0.001	1.08 (1.01, 1.16)	0.021	1.06 (0.99, 1.14)	0.084	1.24 (1.14, 1.33)	<0.001	1.24 (1.13, 1.36)	<0.001
Occupational Physical Activity (OPA)																
No	Ref		Ref		Ref		Ref		Ref		Ref		Ref		Ref	
Yes	1.45 (1.15, 1.83)	0.001	1.43 (1.10, 1.86)	0.007	0.83 (0.67, 1.02)	0.070	1.09 (0.99, 1.20)	0.079	1.22 (1.13, 1.30)	<0.001	1.26 (1.18, 1.35)	<0.001	1.12 (1.04, 1.21)	0.004	1.07 (0.97, 1.18)	0.157

**Table 4:** Results of the adjusted model examining associations between PA domains and changes in health behaviors and mental health outcomes.

	Change in Smoking ^1^ (*n* = 1426)		Change in Drinking ^1^ (*n* = 1923)		Change in Eating ^1^ (*n* = 2576)		Mask-Wearing ^1^ (*n* = 16,197)		Anxiety ^2^ (*n* = 17,180)		Fear ^2^ (*n* = 17,160)		Panic Purchasing of Food ^1^ (*n* = 17,180)		Panic Purchasing of Personal Protective Equipment ^1^ (*n* = 17,180)	
	OR (95% CI)	*p* Value	OR (95% CI)	*p* Value	OR (95% CI)	*p* Value	OR (95% CI)	*p* Value	OR (95% CI)	*p* Value	OR (95% CI)	*p* Value	OR (95% CI)	*p* Value	OR (95% CI)	*p* Value
Vigorous Physical Activity (VPA)																
No	Ref		Ref		Ref		Ref		Ref		Ref		Ref		Ref	
Yes	1.11 (0.87, 1.42)	0.397	1.42 (1.08, 1.86)	0.011	0.86 (0.69, 1.07)	0.168	0.87 (0.78, 0.96)	0.007	1.31 (1.22, 1.42)	<0.001	1.30 (1.20, 1.40)	<0.001	1.09 (1.01, 1.19)	0.036	1.16 (1.05, 1.29)	0.005
Moderate Physical Activity (MPA)																
No	Ref		Ref		Ref		Ref		Ref		Ref		Ref		Ref	
Yes	1.12 (0.88, 1.41)	0.356	1.09 (0.83, 1.43)	0.517	1.27 (1.02, 1.58)	0.034	1.28 (1.16, 1.42)	<0.001	1.33 (1.24, 1.43)	<0.001	1.38 (1.28, 1.48)	<0.001	1.15 (1.07, 1.25)	<0.001	1.24 (1.12, 1.37)	<0.001
Light Physical Activity (LPA)																
No	Ref		Ref		Ref		Ref		Ref		Ref		Ref		Ref	
Yes	0.80 (0.61, 1.06)	0.115	1.01 (0.71, 1.43)	0.958	1.00 (0.77, 1.30)	0.997	1.18 (1.06, 1.33)	0.004	1.34 (1.22, 1.46)	<0.001	1.39 (1.27, 1.51)	<0.001	1.26 (1.14, 1.38)	<0.001	1.15 (1.02, 1.29)	0.023
Total Physical Activity (TPA)																
No	Ref		Ref		Ref		Ref		Ref		Ref		Ref		Ref	
Yes	1.22 (0.87, 1.70)	0.251	1.60 (1.00, 2.55)	0.048	1.23 (0.90, 1.68)	0.196	1.28 (1.12, 1.46)	<0.001	1.42 (1.28, 1.57)	<0.001	1.49 (1.35, 1.65)	<0.001	1.31 (1.17, 1.46)	<0.001	1.25 (1.09, 1.43)	0.001
Leisure Physical Activity (LePA)																
No	Ref		Ref		Ref		Ref		Ref		Ref		Ref		Ref	
Yes	0.77 (0.60, 0.98)	0.032	0.72 (0.55, 0.95)	0.022	1.25 (1.01, 1.54)	0.037	1.19 (1.08, 1.31)	0.001	1.15 (1.07, 1.24)	<0.001	1.14 (1.06, 1.22)	<0.001	1.27 (1.17, 1.37)	<0.001	1.24 (1.12, 1.36)	<0.001
Occupational Physical Activity (OPA)																
No	Ref		Ref		Ref		Ref		Ref		Ref		Ref		Ref	
Yes	1.30 (1.01, 1.67)	0.039	1.31 (0.99, 1.74)	0.055	0.81 (0.65, 1.01)	0.063	1.02 (0.92, 1.13)	0.714	1.20 (1.11, 1.29)	<0.001	1.24 (1.15, 1.33)	<0.001	1.02 (0.94, 1.11)	0.649	1.03 (0.93, 1.14)	0.531

^1^ Adjusted by age, gender, education level, marital status, residency, anxiety, chronic disease, and self-reported health. ^2^ Adjusted by age, gender, education level, marital status, residency, chronic disease, and self-reported health.

## Data Availability

The data used in this study were obtained from the publicly available China Health and Retirement Longitudinal Study (CHARLS) database, which is hosted by the National School of Development at Peking University. The CHARLS dataset is accessible to researchers through an application process to ensure compliance with privacy and ethical considerations. Researchers can request access to the data at http://charls.pku.edu.cn/en (accessed on 16 November 2024).
